# Functional characterization of the ectopically expressed olfactory receptor 2AT4 in human myelogenous leukemia

**DOI:** 10.1038/cddiscovery.2015.70

**Published:** 2016-01-25

**Authors:** S Manteniotis, S Wojcik, P Brauhoff, M Möllmann, L Petersen, JR Göthert, W Schmiegel, U Dührsen, G Gisselmann, H Hatt

**Affiliations:** 1 Department of Cell Physiology, Ruhr-University Bochum, Bochum, Germany; 2 Department of Hematology, University Hospital Knappschaftskrankenhaus Bochum, Bochum, Germany; 3 Department of Hematology, University Hospital Essen, Essen, Germany

## Abstract

The olfactory receptor (OR) family was found to be expressed mainly in the nasal epithelium. In the last two decades members of the OR family were detected to be functional expressed in different parts of the human body such as in liver, prostate or intestine cancer cells. Here, we detected the expression of several ORs in the human chronic myelogenous leukemia (CML) cell line K562 and in white blood cells of clinically diagnosed acute myeloid leukemia (AML) patients by RT-PCR and next-generation sequencing. With calcium-imaging, we characterized in greater detail the cell biological role of one OR (OR2AT4) in leukemia. In both cell systems, the OR2AT4 agonist Sandalore-evoked strong Ca^2+^ influx via the adenylate cyclase-cAMP-mediated pathway. The OR2AT4 antagonist Phenirat prevented the Sandalore-induced intracellular Ca^2+^ increase. Western blot and flow cytometric experiments revealed that stimulation of OR2AT4 reduced the proliferation by decreasing p38-MAPK phosphorylation and induced apoptosis via phosphorylation of p44/42-MAPK. Furthermore, Sandalore increased the number of hemoglobin-containing cells in culture. We described for the first time an OR-mediated pathway in CML and AML that can regulate proliferation, apoptosis and differentiation after activation. This mechanism offers novel therapeutic options for the treatment of AML.

Despite improvements in supportive care and allogeneic transplantation, acute myeloid leukemia (AML) is cured in <50% of patients younger than 60 years old and <20% of patients older than 60 years old.^1^ The treatment’s mainstay is the 3+7 regimen (daunorubicin and cytarabine), which has been in use for >30 years.^1^ Targeted therapies are limited to molecularly defined subtypes of the disease. Although their scientific rationale might be convincing, the treatment results thus far have been disappointing.^2^ There is an urgent need for novel treatment approaches.

Over the last two decades, gene expression analysis has discovered that olfactory receptors (OR) expression is not restricted to the nasal epithelium, but is spread to different parts of the human body.^3–7^ The prostate-specific G protein-coupled receptor (PSGR), also known as OR51E2, is highly expressed in prostate cancer cells and in the prostate cancer cell line LNCaP.^8^ In LNCaP and primary cancer cells *β*-ionon elicits a rapid Ca^2+^ increase by activating the PSGR and inhibits proliferation by MAPK phosphorylation.^6^ Recently, a study showed that in the Huh7 hepatocellular carcinoma cell line, OR1A2 activation increased intracellular Ca^2+^ and reduced cell proliferation.^9^ In enterochromaffin cells, odor stimulation released serotonin in the gut.^10^

K562 is a chronic myelogenous leukemia (CML) cell line derived from a 53-year-old CML-patient in the blast crisis.^11^ The *bcr*-*abl* fusion gene produces a constitutive active tyrosine kinase and leads to an uncontrolled proliferation of undifferentiated blood cells. Unfortunately, tyrosine kinase inhibitors such as imatinib are only effective against CML in newly and in-time diagnosed patients.^12^ In later disease states of CML, a resistance against *bcr*-*abl* tyrosine kinase inhibitor can occur and hinders the treatment of CML.^13^ The later disease state of CML is similar to AML which, if remains untreated, leads to death within a few weeks. Until today, mechanism besides the *bcr*-*abl* pathways are widely unknown, but can provide a novel therapeutic target for the treatment against later stages of resistant CML and AML.

We detected seven ORs that were expressed (>1 FPKM) in K562, and confirmed their expression in white blood cells of newly diagnosed AML patients. We focused on the functional characterization of OR2AT4, because the ligand Sandalore and the antagonist Phenirat are already described and enable therefore further investigations.^14^ We characterized the OR2AT4-mediated signaling pathway and observed alternations in the proliferation, apoptosis and erythrocyte differentiation caused by the regulation of MAPK phosphorylation.

## Results

### K562 and white blood cells of AML patients express olfactory receptors

**Figure 1:**
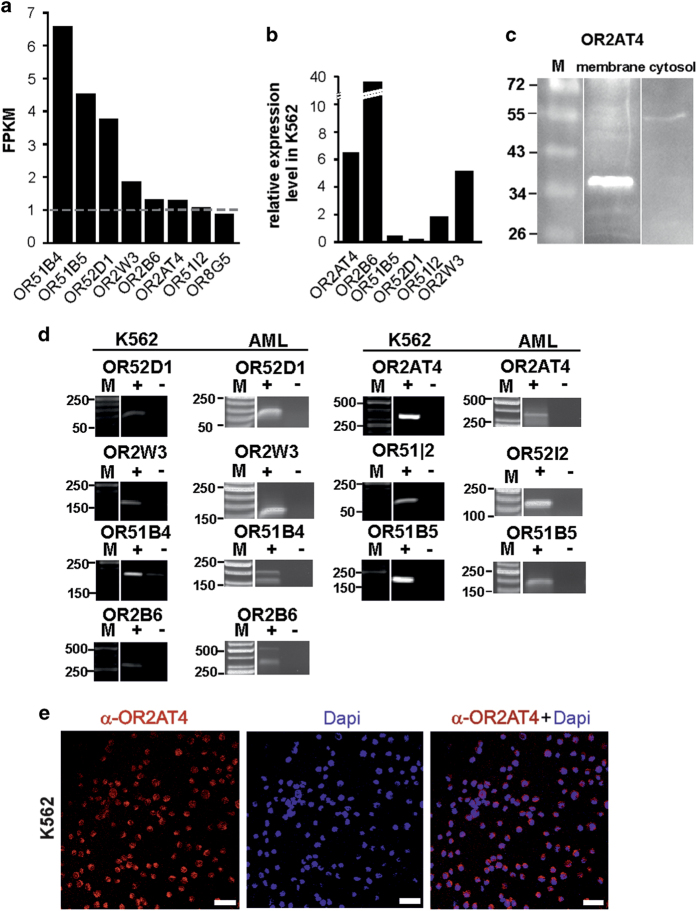
ORs are expressed in the K562 and in the white blood cells of AML patients. (**a**) K562 RNA-Seq data revealed 7 ORs expressed (>1 FPKM). (**b**) With qPCR experiements we compared the expression strength of ORs in K562 to ORs in AML-patient blood. Some ORs, such as the OR2AT4, were higher expressed in AML than in K562 (*n*=3). (**c**) The OR2AT4 protein (35 kDa) is detected in the K562 membrane but not in the cytosolic fraction. (**d**) We confirmed the expression of the ORs in K562 and AML-patient blood by RT-PCR. Same ORs were found in cell types. (**e**) Immunofluorescence staining of OR2AT4 in K562 cells was analyzed with a confocal microscope. Anti-OR2AT4, red; DAPI, blue; scale bars, 20 μm.

For the initial detection of ectopically expressed ORs in myelogenous leukemia, we reanalyzed a free online available NGS data set from the CML cell line K562 (SRR1207231). In total, the expression of 7 ORs >1 FPKM could be shown (Figure 1a). In Figure 1b we compared the expression strength of ORs in K562 cells between ORs in AML-patient blood cells. Our results showed that most of the ORs detected >1 FPKM were higher expressed in all tested AML-patient blood samples. Our western blot analysis additionally verified the expression of the OR2AT4 protein in membrane components of K562 cells (Figure 1c). We validated by RT-PCR in K562 cDNA and in white blood cells of AML patients’ cDNA the expression of the ORs (Figure 1d). One of the highest expressed ORs in K562 and AML patients’ cDNA was the OR2AT4. Immunocytochemical staining of OR2AT4 showed its expression in K562 cells (Figure 1e). In addition, we investigated the expression of members of the common G-protein-coupled signaling pathways (Supplementary Figure 1). Here, we detected the expression of different adenylate cyclases, G-proteins, protein lipase C (PLC) and PKA.

### Sandalore increases the intracellular Ca^2+^ level and the cAMP concentration in K562 cells

**Figure 2:**
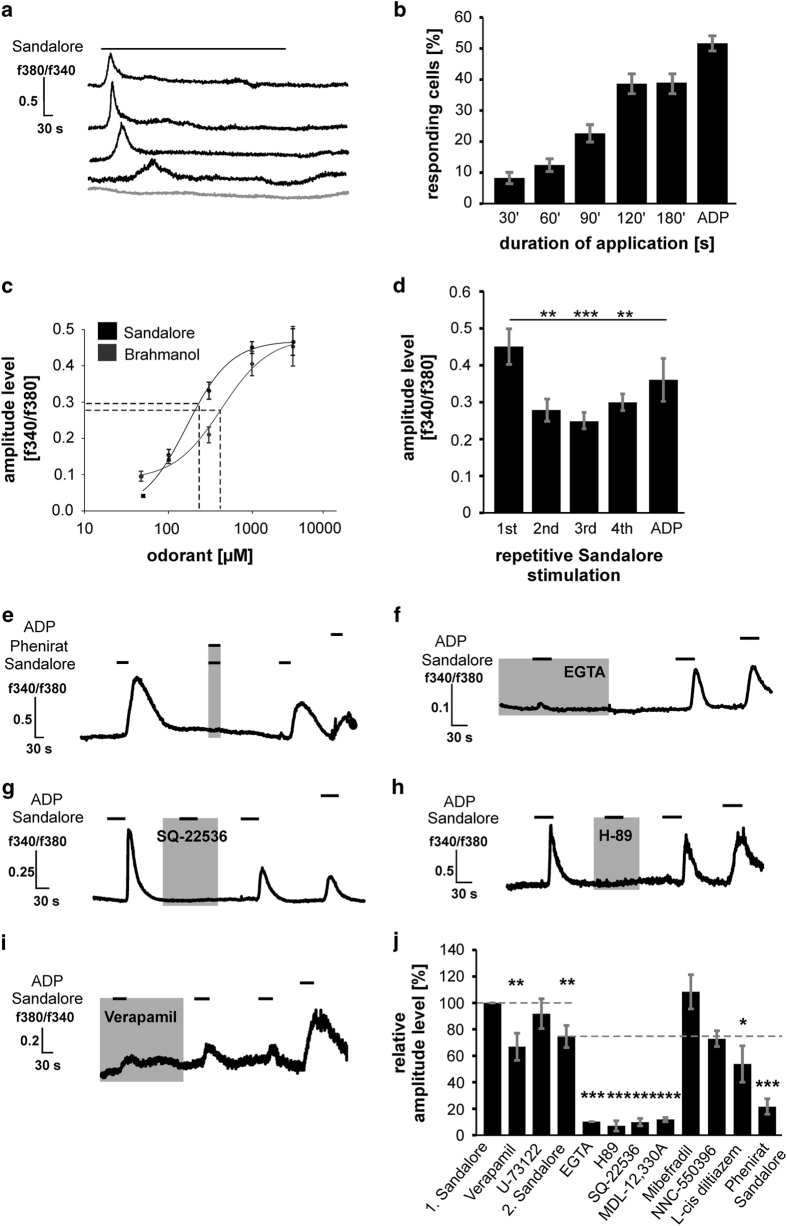
Sandalore increases intracellular Ca^2+^ concentrations in K562 cells. (**a**) In long-term experiments 300 μM Sandalore increased the intracellular Ca^2+^ levels (blue lines; *n*=383). Ringer application had no effect (gray line). (**b**) We observed a time-dependent activation rate during long-term stimulation. (**c**) Sandalore: EC_50_=265 μM (±32; *n*=684), Brahmanol EC_50_=392 μM (±29*; n*=380). (**d**) Receptor response desensitization was observed after repetitive Sandalore stimulation (~0.1 f340/f380). (**e**) Phenirat blocked the Sandalore-induced increase in intracellular Ca^2+^ (*n*=55). (**f**) 10 mM EGTA showed that the Sandalore-induced increase of intracellular Ca^2+^ is dependent on extracellular Ca^2+^ (*n*=85). (**g**) SQ-22536 (10 μM, *n*=201) blocked the Sandalore-induced increase in intracellular Ca^2+^. (**h**) 10 μM H-89 blocked the Sandalore-induced increase in intracellular Ca^2+^ (*n*=78). (**i**) 20 μM Verapamil significantly decreased the Sandalore-evoked increase in intracellular Ca^2+^ (*n*=68). (**j**) Inhibitor experiments were analyzed relative to the first and second application of Sandalore. Other inhibitors used in these experiments can be found in our Supplementary Data.

To investigate whether Sandalore could activate the OR2AT4 in K562 cells, long-term (10 min) calcium-imaging experiments with 300 μM Sandalore were performed and revealed that the cells increased temporary their intracellular Ca^2+^ level within the first 2 min during odor application (Figure 2a). The number of Sandalore-responding cells increased during the time for which the odorant was applied. In the first 30 s, ~8% of the cells increased their intracellular Ca^2+^ level (Figure 2b). After a 2 min application of Sandalore, ~40% of the cells increased their intracellular Ca^2+^ concentration. Dose-response curves for the OR2AT4 agonist Sandalore and for the weaker agonist Brahmanol showed that 50 μM was the lowest concentration that could slightly increase the intracellular Ca^2+^ level (Figure 2c). Saturation was reached for both substances at 1 mM. The calculated EC_50_ for Sandalore was 265 μM (±32), and that for Brahmanol was 392 μM (±29; Supplementary Figure 2a). We also observed desensitization of the Ca^2+^ response after repetitive Sandalore stimulation (Figure 2d).

Next, we performed pharmacological experiments to analyze the signaling pathway of the OR2AT4. To validate the involvement of OR2AT4 in the Sandalore-evoked increase in intracellular Ca^2+^, we used the OR2AT4 antagonist Phenirat^14^ (Figure 2e). By coapplying Phenirat and Sandalore, Ca^2+^ increase could be completely blocked in a reversible manner.

Next we determined the source of Ca^2+^ using the Ca^2+^ chelator EGTA. The absence of extracellular Ca^2+^ abolished the Sandalore-evoked increase in intracellular Ca^2+^ (Figure 2f). To investigate the induction of a cAMP-mediated pathway in K562 cells, we inhibited the adenylate cyclase pathway with SQ-22536 and MDL-12,330A (Figure 2g, Supplementary Figure 2d). Both inhibitors reversibly abolished the Sandalore-evoked Ca^2+^ increase. According to this, cAMP-Glo Assay (Promega, Madison, WI, USA) experiments validated the Sandalore-evoked increase in cAMP after odor incubation (Supplementary Figures 3a and b). In addition, the PKA inhibitor H-89 abolished the Sandalore-evoked Ca^2+^ increase (Figure 2h). We show that the irreversible PLC inhibitor U-73122 could not inhibit the Sandalore-evoked increase in intracellular Ca^2+^ (Supplementary Figure 2c).

To observe whether calcium channels were activated downstream, we tested the T-type channel blockers NNC-55396 and mibefradil (Supplementary Figures 2d and e). Neither inhibitors could inhibit the Sandalore-induced Ca^2+^ increase. However, L-cis diltiazem, an inhibitor of cyclic nucleotide-gated channels, which additionally inhibit L-type Ca^2+^ channels, reduced the Sandalore response significantly (Supplementary Figure 2f). To investigate whether a L-type voltage-gated Ca^2+^ channel was involved, we used the inhibitor Verapamil (Figure 2i). Cells incubated in Verapamil had a significantly smaller increase in intracellular Ca^2+^. An overview of the calcium-imaging experiments is shown in Figure 2j.

Owing to the observed desensitization effect that we showed before, we investigated the significance of the blocking effect, while comparing the Ca^2+^ increase evoked by Sandalore and the inhibitors to the average first and second Sandalore application without inhibitors (Figure 2d). The amplitudes of preincubated inhibitors U-73122, EGTA and Verapamil were compared with the first control application of Sandalore.

### Sandalore evokes an increase in intracellular Ca^2+^ in white blood cells from AML patients

**Figure 3:**
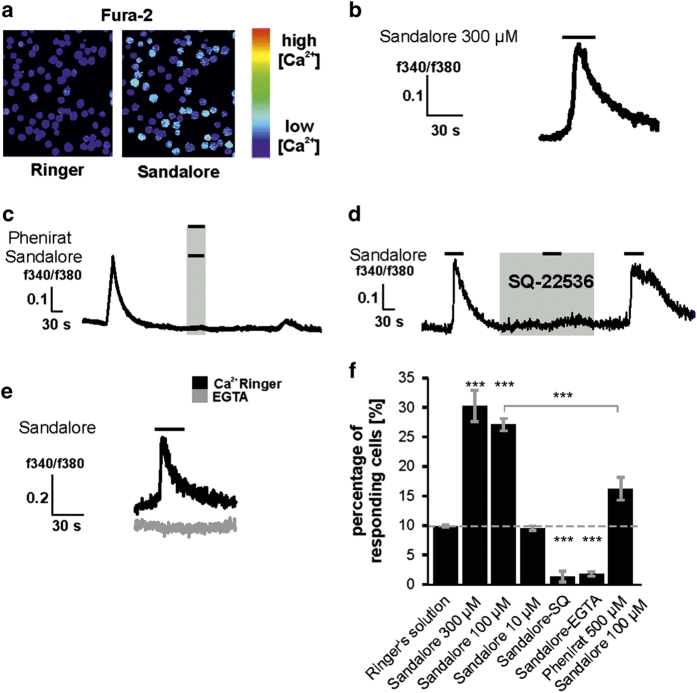
Sandalore increases the intracellular Ca^2+^ in the white blood cells of AML patients. (**a**) White blood cells from AML patients increased their intracellular Ca^2+^ during Sandalore application. (**b**) The OR2AT4 agonist Sandalore increased the intracellular Ca^2+^ level (*n*=6). (**c**) The OR2AT4 antagonist Phenirat abolished the Sandalore-evoked increase in intracellular Ca^2+^ (*n*=5). (**d**) SQ-22536 (10 μM) completely blocked the Sandalore-induced increase in intracellular Ca^2+^ (*n*=3). (**e**) 10 mM EGTA-incubated cells were not able to increase the amount of intracellular Ca^2+^ during Sandalore application (gray line, *n*=4). (**f**) Phenirat, SQ-22536 and EGTA significantly reduced the number of responding cells.

To investigate whether Sandalore could also increase the intracellular Ca^2+^ in blood cells of sickened leukemia patients, such as it did in K562 cells, we isolated the white blood cells of clinically newly diagnosed AML patients and measured them with calcium-imaging. During application of Sandalore, AML cells significantly increased their intracellular Ca^2+^ (Figures 3a and b). The Sandalore-induced increase in intracellular Ca^2+^ could be blocked by co-treatment with the OR2AT4 antagonist Phenirat (Figure 3c). Both, extracellular Ca^2+^ and adenylate cyclase were necessary for the increase in intracellular Ca^2+^ in white blood cells of AML patients (Figures 3d and e). However, in contrast to K562 cells, many AML cells spontaneously showed transient Ca^2+^ signals. To distinguish between spontaneous and odorant-induced increases we applied Ringer’s solution to determine the rate of responding cells during application. On average, 10% (±0.2) of the cells responded spontaneously during the application of Ringer’s solution (Figure 3f). Nevertheless, a significantly greater number of cells increased in intracellular Ca^2+^ during the application of 100–300 μM Sandalore (27–31%).

### Sandalore increases p44/42-MAPK and Akt phosphorylation and induces time-dependent increases and decreases in p38-MAPK phosphorylation

**Figure 4:**
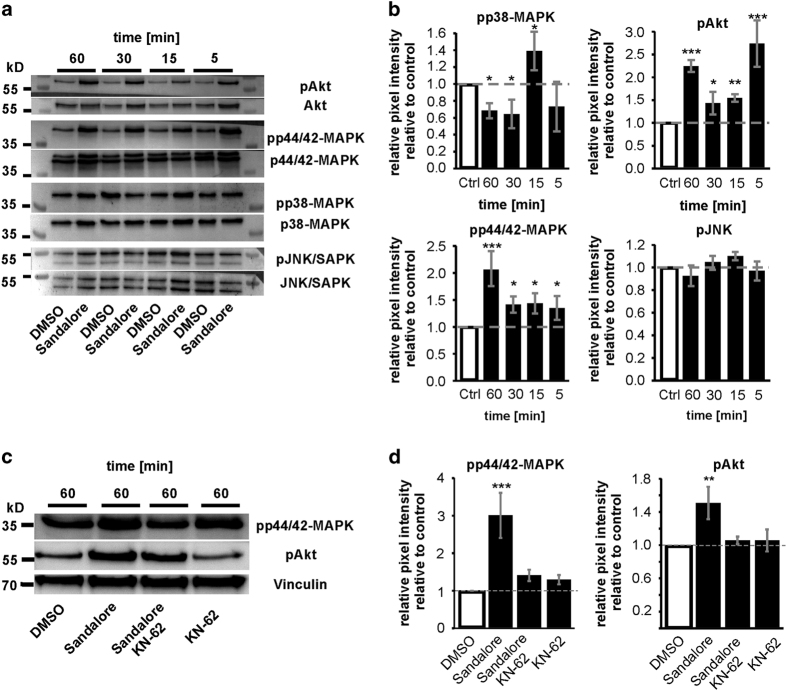
Sandalore induces Akt and p44/42-MAPK phosphorylation and decreases p38-MAPK phosphorylation. (**a**) Exemplary blots for the alternation of the MAPK phosphorylation during Sandalore incubation. (**b**) The Sandalore-induced phosphorylation of MAPKs and Akt (*n*=14). (**c**) The inhibition of CaMKII significantly decreased the Sandalore-evoked p44/42-MAPK and Akt phosphorylation. Vinculin was used as a further loading control. (**d**) KN-62 significantly decreased p44/42-MAPK and Akt phosphorylation (*n*=6).

To observe how the increase in intracellular Ca^2+^ caused by Sandalore affected the main physiological functions in K562 cells, such as differentiation, proliferation or apoptosis, we performed western blot experiments to investigate the phosphorylation of different kinases. Sandalore increased the p44/42-MAPK phosphorylation 2.1 (±0.22) times higher, and the Akt phosphorylation 2.7 (±0.34) times higher compared with the untreated cells (Figures 4a and b). p38-MAPK phosphorylation was reduced significantly by ~0.74 (±0.09) after 60 min, except in the 15 min probe, in which p38-MAPK phosphorylation was significantly increased (1.39±0.21). The phosphorylation of JNK-SAPK did not alter. Previous studies have shown that intracellular Ca^2+^ could bind to calmodulin and activate calmodulin kinase 2 (CaMKII), which is able to trigger the phosphorylation of different MAPKs.^15,16^ Therefore, cells were treated for 1 h with 100 μM Sandalore, including 10 μM of the CaMKII inhibitor KN-62 (Figures 4c and d). The CaMKII inhibitor KN-62 and Sandalore-treated cells showed decreased p44/42-MAPK and Akt phosphorylation, thus confirming the initiation of phosphorylation by Sandalore, and the activation of OR2AT4 evoked increased intracellular Ca^2+^.

### The activation of OR2AT4 by Sandalore inhibits cell proliferation in K562 by decreasing the early phosphorylation of p38-MAPK

**Figure 5:**
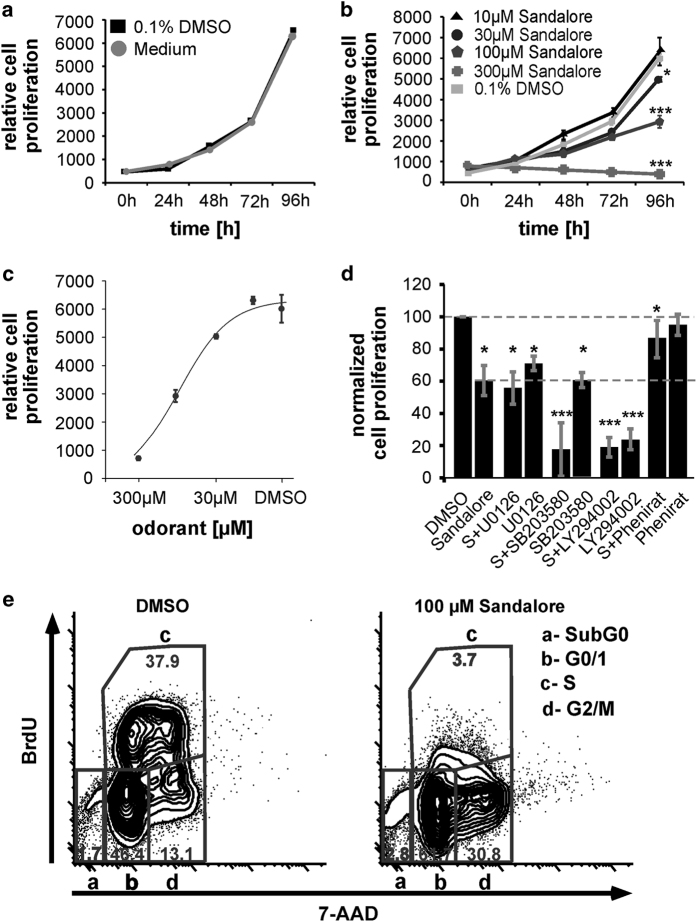
Sandalore decreases the K562 proliferation. (**a**) 0.1% DMSO-treated cells were compared with the proliferation in RPMI-1640 medium incubated cells (*n*=3). (**b**) Relative proliferation for Sandalore-treated cells (*n*=32). (**c**) Dose-response curve for the inhibitory effect of Sandalore on the proliferation (IC_50_=74 μM). (**d**) Pharmacological characterization for the Sandalore-induced inhibition of proliferation (*n*=5-9). (**e**) Effect of Sandalore on cycle progression. Cells were analyzed after 5 days of incubation with 0.1% DMSO or 100 μM Sandalore (*n*=6).

Sandalore was able to decrease the cell proliferation significantly in a dose-dependent manner (Figures 5a and b). Sandalore (100 μM) inhibited proliferation by ~54%, 300 μM Sandalore inhibited the proliferation completely. The IC_50_ for Sandalore was 74 μM (Figure 5c). To investigate the involvement of MAPKs and Akt, we incubated the cells with the appropriate inhibitors (Figure 5d). All MAPK-inhibitors decreased the proliferation, indicating the involvement of the Sandalore-evoked reduction in p38-MAPK phosphorylation in this process. Phenirat was used to investigate whether the antagonist could prevent the inhibitory effect of Sandalore (Figure 5d). Incubation with Phenirat and Sandalore (1 : 1) could significantly increase again the number of proliferating cells to the control level.

To study the anti-proliferative effect of Sandalore in greater detail, we analyzed K562 cell cycle progression after 5 days of culture. The anti-proliferative effect of Sandalore was due to a 30% decrease of cells in the S-phase of the cell cycle. In contrast, the proportion of cells in the G0/1- and G2/M-phases was highly increased (Figure 5e).

### The activation of OR2AT4 by Sandalore evokes cell apoptosis in K562 by increasing p44/42-MAPK phosphorylation

**Figure 6:**
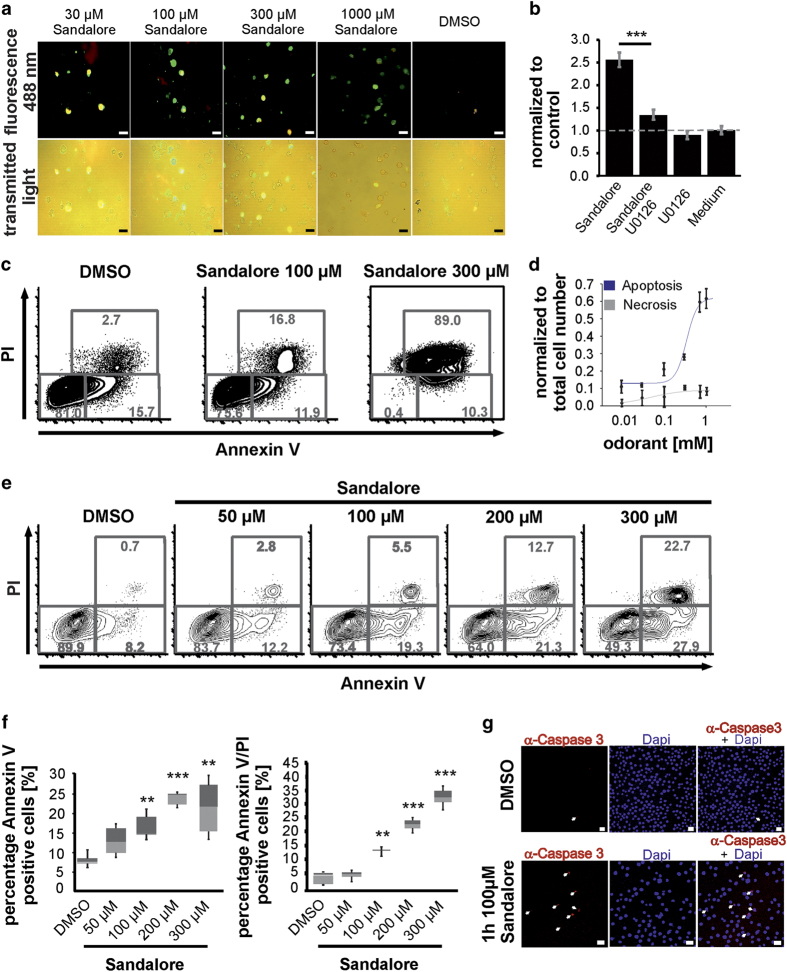
Sandalore evokes apoptosis and activates caspase-3. (**a**) K562 cells were incubated for 1 h with 30 μM–1 mM Sandalore. Sandalore induce apoptosis (green*; n*=45). (**b**) Inhibition of p44/42-MAPK phosphorylation during Sandalore incubation abolished the induction of apoptosis (*n*=5). (**c**) Analysis of K562 apoptosis and cell death by Annexin-V and 7-AAD staining after 5 days of incubation (*n*=9). (**d**) Sandalore increased the apoptosis rate, IC_50_=395 μM (*n*=45). (**e**) Apoptosis in primary AML-patient blood cells after 4 h incubation (*n*=15). (**f**) Box plots for the Sandalore-induced early apoptosis (PI-, Annexin-V+) and late apoptosis (PI+ and Annexin-V+) in primary AML-patient blood cells (*n*=15). (**g**) 1 h incubation with 100 μM Sandalore activates the caspase-3. Scale bar, 20 μm.

To investigate whether Sandalore can induce apoptosis, we used the Annexin-V/PI (propidium iodide) Assay (Biovision, Milpitas, CA, USA) under microscopic conditions. The number of apoptotic cells increased when higher concentrations of Sandalore were used (Figure 6a). To investigate the extent to which the phosphorylation of the MAPKs and Akt was involved in Sandalore-evoked apoptosis, we used MAPK-inhibitors (Figure 6b, Supplementary Figure 4). By blocking p44/42-MAPK phosphorylation, the apoptotic influence of 300 μM Sandalore was completely abolished.

Finally, we confirmed the induction of K562 apoptotic cell death by flow cytometry using Annexin-V in combination with 7-AAD staining. Although 100 μM Sandalore lead to a doubling of the apoptotic cell death rate, 300 μM almost depleted all of the viable cells (Figure 6c). The IC_50_ of Sandalore-induced apoptosis was 395 μM (Figure 6d).

To study whether Sandalore was capable of inducing apoptosis of primary leukemic cells, we performed cultures with peripheral blood blasts of AML patients. Remarkably, Sandalore induced the apoptotic cell death of primary AML cells in culture in a concentration-dependent manner (Figures 6e and f). Further immunocytochemical staining revealed the activation of caspase-3 after 1 h of treatment with 100 μM Sandalore, compared with the control 0.1% DMSO (Figure 6g).

### Sandalore enhances the hemoglobin synthesis of K562 cells by increasing p44/42-MAPK and time-dependent p38-MAPK phosphorylation

**Figure 7:**
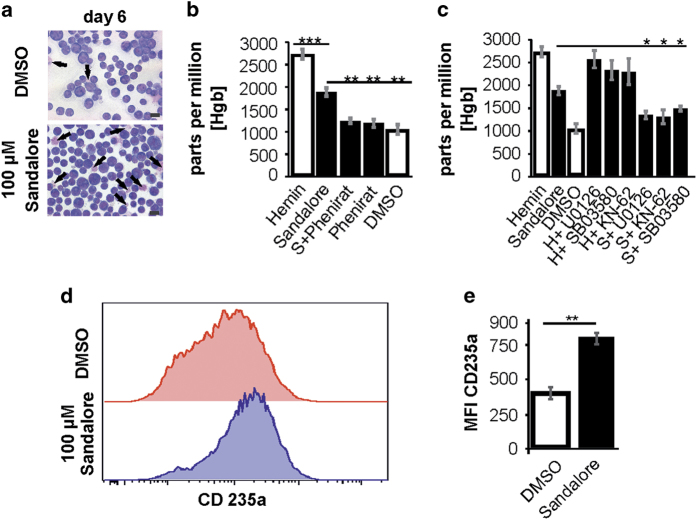
Sandalore enhances the hemoglobin synthesis of K562 cells and the number of pro-erythroblasts. (**a**) Pappenheim staining revealed a greater number of hemoglobin-carrying cells in the Sandalore-incubated culture (blue arrows). Scale bars, 10 μm. (**b**) Sandalore (100  μM) significantly enhanced the hemoglobin synthesis of K562 cells after 6 days of incubation. 100 μM Phenirat prevented the Sandalore-increase of the hemoglobin production (*n*=9). (**c**) p44/42-MAPK phosphorylation and the early stage phosphorylation of p38-MAPK (minute 15) are involved in the hemoglobin synthesis enhancement through Sandalore (*n*=9). (**d**) Erythroid differentiation was analyzed by staining the terminal erythroid marker CD235a (glycophorin A). DMSO and Sandalore cultures at 10 days (*n*=3). (**e**) The median fluorescence intensity (MFI) of the CD235a staining in K562 cells (0.1% DMSO, 100 μM Sandalore) was quantified (*n*=3).

The altered MAPK-phosphorylation induced by 100 μM Sandalore was able to initiate cell differentiation (Figure 7a). Here, Sandalore-treated cells shared a greater number of differentiated cells. To investigate whether the red cells were hemoglobin-carrying cells, we used the Hemoglobin Assay (Figures 7b and c). These spectrometric analysis showed that the amount of hemoglobin (HgB) concentration was significantly greater after a 6-day treatment with 100 μM Sandalore (Figure 7b). As a positive control for the enhancement of HgB synthesis we used hemin.^17^ Sandalore-treated cells almost doubled in the number of hemoglobin-carrying cells. Thus, the OR2AT4 antagonist Phenirat significantly decreased Sandalore-induced hemoglobin synthesis to the level of the control cells and verified the involvement of OR2AT4. In contrast, 100 μM Phenirat alone did not influence the HgB production.

To examine the involvement of MAPKs, Akt and CaMKII, we used the same inhibitors as described before (Figure 7c). The strong observed phosphorylation of p44/42-MAPK and the early (minute 15) p38-MAPK phosphorylation initiated by 100 μM Sandalore were both necessary for the induced HgB production. According to this, inhibition of CaMKII also prevented hemoglobin synthesis. To quantify Sandalore-induced erythroid differentiation of K562 cells, we stained for the terminal erythroid marker CD235a. In Sandalore-treated cultures the majority of cells expressed high levels of CD235a, whereas in DMSO control cultures there was still a high proportion of cells expressing low levels of CD235a (Figures 7d and f). The median fluorescence intensity of CD235a expression by Sandalore-treated cells is increased by 50% (Figure 7f).

## Discussion

Several studies have described the anti-cancer effects induced by odorous substances, often terpenes, which are components of essential oils.^9,18–21^ Half of all produced anti-cancer drugs are natural products or synthetic derivatives.^22,23^

We used K562 cells, a common cell model for investigating the physiology of CML, and primary cells of newly detected AML patients owing to their high number of blastic cells. A previous study characterized OR2AT4 in transfected HEK293 cells, the HaCaT cutaneous cell line and primary keratinocytes determining the specific agonists Sandalore, Javanol and Brahmanol and the antagonists Phenirat and Oxyphenilon.^14^ Our experiments showed that, in both K562 and AML cells, Sandalore activated the OR2AT4 by a cAMP-mediated pathway, via L-type calcium channels, which contribute to the influx of extracellular Ca^2+^ downsteam. To distinguish between spontaneous and the odor-induced activation we performed control experiments in which we repetitively applied Ringer’s solution. Also in native AML cells, extracellular Ca^2+^ and adenylate cyclase were necessary for the Sandalore-induced increase in intracellular Ca^2+^. According to this finding, other ectopically expressed ORs, such as the OR1A2 in the Huh7 hepatocarcinoma cell line, has been described to activate a cAMP-dependent signaling pathway.^9^ Calcium-imaging based dose-response curves showed that the EC_50_ for the activation of OR2AT4 by Sandalore in K562 (265 μM) was in a similar concentration range as previously demonstrated for ORs in keratinocytes (430 μM).^14^ In addition, the EC_50_ value for the cAMP production by Sandalore in K562 (310 μM) was similar to that observed in keratinocytes (197 μM).^14^

Sandalore inhibited the proliferation of K562 cells by preventing the p38-MAPK phosphorylation. In contrast, the activation of OR2AT4 in keratinocytes was shown to increase the cell proliferation by up to ~20%.^14^ Comparing both cell systems, one reason for this difference could be the different p38-MAPK phosphorylation induced by Sandalore. In keratinocytes, p38-MAPK was significantly increased,^14^ while in contrast, we observed decreased p38-MAPK phosphorylation in K562 cells. According to this findings, other studies of K562 cells showed that an increase in p38-MAPK did not affect the proliferation of K562 cells, whereas decreased p38-MAPK phosphorylation significantly reduced cell proliferation.^24^ However, the involvement of MAPKs in the regulation of physiological mechanisms vary depending on the cell system.^25,26^

Flow cytometric analyses validated the inhibition of proliferation by Sandalore through significant reduction of the S-phase and growth arrest associated with G2/M phase arrest of cell cycle progression. In other studies, similar mechanisms were shown during the inhibition of K562 proliferation initiated by other substances.^27^

Next, we showed that Sandalore significantly increased the number of early apoptotic cells after 1–4 h of incubation. Inhibitor experiments revealed the involvement of Sandalore-induced increased p44/42-MAPK phosphorylation in this process. Previous studies have shown that for apoptosis both possibilities, namely inhibition and enhancement by p44/42-MAPK phosphorylation, exist, depending on either the substance or the receptor.^28–32^ A growing number of newly performed studies have reported that phosphorylation of p44/42-MAPK within 24 h initiated apoptosis.^33–43^ However, in the present work, we showed that apoptosis induced by Sandalore was evoked by an increase in intracellular Ca^2+^, which activated CaMKII and initiated p44/42-MAPK phosphorylation, followed by caspase-3 activation. Wang *et al.* showed a similar case evoked by a cyclic lipopeptide.^44^ Later, Wang *et al.* observed that this apoptotic process was due to an increase in intracellular Ca^2+^ and increased p44/42-MAPK phosphorylation, followed by Bcl-2 downregulation, cytochrome *c* and caspase-3 activation.^31^ Beside the investigated involvement of CaMKII in activation of MAPK phosphorylation it could be possible that calcium activates also other signaling kinases, such as the PKC and which could enhance thereby the MAPK phosphorylation.

In addition, 100 μM Sandalore could increase the amount of produced hemoglobin in K562 cell culture after 6–10 days of incubation. According to previous studies, inhibiting early p38-MAPK and p44/42-MAPK phosphorylation^43,45–47^ prevented the Sandalore-induced differentiation of K562 cells. Hemin-induced hemoglobin synthesis is, according to other studies, not dependent on the observed MAPK phosphorylation.^46^ These data were supported by our flow cytometric analysis with CD235a, an erythrocytic marker, which revealed a 100% increase in CD235a-carrying cells after Sandalore incubation.

### Potential role of OR2AT4 in the treatment of AML

Our data suggested that OR2AT4 is widely expressed in AML blasts. Stimulation of OR2AT4 by Sandalore led to reduced proliferation and enhanced apoptosis and differentiation, all of which are desired effects of antileukemic drugs. Translating our results into the clinic faces two major problems. First, the concentrations required to inhibit leukemic cells *in vitro* were high, on the order of 300 μM. One way of circumventing the problem of high-Sandalore concentrations would be to develop more powerful OR2AT4 agonists able to elicit effects at lower concentrations. Second, as has been discussed above, the stimulation of OR2AT4 has different effects on different tissues, which could have detrimental effects on the organism. Further preclinical studies are required to determine whether the OR2AT4 signaling pathway might be exploited for the treatment of leukemia.

## Materials and methods

### Cell culture and patient samples

K562 cells were purchased from LGC Standards GmbH (Wesel, Germany). The cells were cultured in RPMI-1640 (Life Technologies, Carlsbad, CA, USA) containing 10% (v/v) fetal bovine serum, 5% GlutaMAX (Life Technologies) with addition of penicillin/streptomycin. The analysis of blood samples from AML patients was approved by the ethics committee of the University Hospital Essen and of the Knappschaftskrankenhaus Bochum (ZOKS 3841-10). The patients provided informed consent according to institutional guidelines. Of all, 25 patients with various types of AML participated in the study. Details of the leukemias’ and the patients’ treatment histories are provided in the Supplementary Table 1. Blood was drawn from central venous lines and was incubated with Lymphoprep (Stemcell Technologies, Cologne, Germany), according to the manufacturer’s instructions. Erythrocytes were eliminated with lysing buffer (BD Biosciences, Heidelberg, Germany), followed by centrifugation.

To measure cell viability, a proliferation assay was performed every 24 h in 96-well plates using the CyQUANT cell proliferation kit (Life Technologies), according to the manufacturer’s protocol. Cells were seeded at a density of 2×10^4^ cells/ml. All of the odorants used were purchased from Sigma Aldrich (Sigma Aldrich, Munich, Germany). For measurement of hemoglobin synthesis, we used the Hemoglobin Assay Kit (Sigma Aldrich). The kit was used as described in the manufacturer’s protocol. We incubated 5 ml of cells at 1×10^6^ cells/ml in cell culture flasks with 100 μM Sandalore for 6 days. Medium and odorant were replaced every second day. To be able to compare the amount of hemoglobin-containing cells, before the spectrometric analysis, control and Sandalore-treated cells were divided into equal numbers of cells using a Neubauer counting chamber. To distinguish between the different cell types in culture, Sandalore-treated cells and untreated cells were stained with the Pappenheim standard staining solution, according to the manufacturer’s protocol (LT-SYS, Berlin, Germany).

### Immunocytochemistry

Experiments were performed essentially as described previously^14^ with the antibodies indicated in the figure legends. For the OR2AT4 same custom-designed antibodies were used against the C-terminus, polyclonal (1 : 50; Eurogentec, Seraing and Belgium) and anti-caspase-3, polyclonal (1:300; Sigma Aldrich) as described elsewhere.^14^

### Calcium imaging

K562 cells and white blood cells from AML patients were incubated for 20 min in concanavalin A-laminated (Sigma Aldrich) 35-mm dishes. Before starting the calcium-imaging experiments, RPMI-1640 medium was replaced with Ringer’s solution. Calcium-imaging experiments were performed as described previously.^7^ To investigate cell viability, 100 μM ADP was applied to the cells at the end of the measurements. In previous studies, ADP has been shown to increase the amount of intracellular Ca^2+^ in K562 cells.^48^

### Flow cytometry

To determine the impact of Sandalore on the cell cycle status of K562 cells, bivariate BrdU/DNA flow cytometric analysis was performed. After 5 days of Sandalore exposure, the cells were pulsed with BrdU for 1 h. Anti-BrdU and 7-AAD staining was completed according to the manufacturer’s instructions (BrdU Flow kit, BD Biosciences).

Annexin-V/PI staining was used to study apoptosis (Annexin-V+/PI-) and apoptotic cell death (Annexin-V+/PI-) in K562 cells and primary AML cells after Sandalore exposure (FITC Annexin-V Apoptosis Detection Kit I, BD Pharmingen). Erythroid differentiation was assayed by staining the K562 cells with anti-CD235a (monoclonal mouse anti-human CD235a glycoporin A/FITC and A/RPE, clone JC159). Flow cytometric data acquisition was performed with a LSRII flow cytometer (BD Bioscience) and the data were analyzed by DIVA (BD Bioscience) and FlowJo software (Treestar, Ashland, OR, USA).

A more detailed description for the methods can be found in the Supplementary Data.

## Abbreviations

AML: acute myeloid leukemia
CML: chronic myeloid leukemia
OR: olfactory receptor
PSGR: prostate specific G protein-coupled receptor
MAPK: mitogen activated protein kinase
PLC: phospho lipase C
CaMKII: calmodulin kinase 2
HgB: hemoglobin
