# Supplementary material for: Functional characterization of the ectopically expressed olfactory receptor 2AT4 in human myelogenous leukemia
Source: Cell Death Discov. 2016 Jan 25;2:15070–. doi: 10.1038/cddiscovery.2015.70 (PMC4979481; doi:10.1038/cddiscovery.2015.70)
Supplement: Supplementary Information [file cddiscovery201570-s1.doc]

***Supplementary Information***

***Supplementary Material and Methods:***

***Cell proliferation.*** Cells were seeded in cell culture flasks at a density of 1 x 10^5^ cells/mL and were treated with the different odorant concentrations (10 µM - 300 µM) or with 0.1% DMSO as a control. Alternatively, the cells were stimulated with odors, including inhibitors of p38-MAPK, Akt, and p44/42-MAPK. To measure cell viability, a proliferation assay was performed every 24 h in 96-well plates using the CyQUANT cell proliferation kit (Life Technologies), according to the manufacturer’s protocol. The proliferation was investigated for 5 days

***PCR experiments.*** RNAs of K562 cells and the white blood cells of AML patients were isolated using the QIAamp RNA Blood Mini Kit (Qiagen, Hilden, Germany), as described in the manufacturer’s instructions. After isolating the RNA, we used the TURBO DNA free Kit (Life Technologies, Carlsbad, CA, USA) to prevent contamination of genomic DNA. cDNA synthesis was performed using the iScriptTM cDNA Synthesis Kit (BioRad Laboratories, Hercules, CA, USA). As a control for possible contamination with genomic DNA, we performed a (-) RT-control. All PCR-experiments were performed using GoTaq qPCR Master Mix (Promega, Madison, WI, USA) (20 µl) and OR-specific primer pairs (10 pmol). The RT- and qPCR amplifications were performed for 40 cycles (1 min, 95 °C; 45 s, 59 °C; 1 min, 72 °C). The housekeeping gene TBP was used as a positive control. PCR was performed with cDNA (equivalent to ~30 ng total RNA) and specific primer pairs. PCR was performed using the Mastercycler realplex^2^ (Eppendorf, Hamburg, Germany), and the resulting products were confirmed using Sanger sequencing. All of the experiments were conducted in triplicate. TBP was used as housekeeping gene in our qPCR experiments. The following primer pairs were constructed:

OR2AT4 forward: 5’ GCCCATCCCAGCAGTAGTAAG 3’;

OR2AT4 reverse: 5’ CTCACACCAATTCTCAACCCCCTC 3’;

OR51B5 forward: 5’ CAATGGCACCCTCCTTCTTC 3’;

OR51B5 reverse: 5’ CAAGCAGAATGCCAGACTCG 3’;

OR52D1 forward: 5’ TCTGAGTACCTGTGGCTCCCAC 3’;

OR52D1 reverse: 5’ CGGTGGGTGAGAAGGAGAAGA 3’;

OR2W3 forward: 5’ CTGCCGGGGCTTGGTGTCAG 3’

OR2W3 reverse: 5’ TCCACCTCGTGGTGCCCACA 3’

OR52l2 forward: 5’ GTGGCCTTCATTGCTGCCTCCTA 3’

OR52l2 reverse: 5’ CACTACATCCTGCCCCAACCAGG 3’;

OR51B4 forward: 5’ ATTGCCATCCGCACACACCACTGAG 3’;

OR51B5 reverse: 5’ CCAGACGCAATGCCCATCACTGT 3’;

OR2B6 forward: 5’ TCGTGGCTGTGTAGCCCAGC 3’;

OR2B6 reverse: 5’ GCAGCTGCCAACTGGAGGGCA 3’;

TBP forward: 5’ GGGGAGCTGTGATGTGAAGT 3’;

TBP reverse: 5’ CCAGGAAATAATTCTGGCTCA 3’.

***Next-generation sequencing.*** The next-generation sequencing (NGS) data for K562 cells, sequenced with an Illumina HiSeq2000 Genome Analyzer, was obtained from the NCBI GEO database (Acc. No: SRR1207231). We reanalyzed the sequencing data using TopHat and Cufflinks software^1^ on a Linux-based system, as described in previous studies^2^. The reads were mapped onto the human reference genome (hg19), FPKM values were calculated using the RefSeq gene model (UCSC Genome Bioinformatics, University of California Santa Cruz), and the expression of 13952 genes was detected (< 1 FPKM). One FPKM represents the number of fragments per kilobase of an exon mapped per million reads^1^.

***cAMP-Glo^TM^ Assay.*** To determine the intracellular cAMP level of odorant-stimulated cells, we used cAMP-Glo^TM^ Assay (Promega) according to the manufacturer’s instructions. Before starting the experiments, we generated a cAMP standard curve to compare the strength of luminescence signals with the different cAMP concentrations, as described elsewhere^3^. The K562 cell suspension was cultured in a flask as previously described. During the experiments, cells were placed in 96-well plates at a density of 5 x 10^5^ cells/well and were treated with different odorants at 1 mM concentration for 5 min at room temperature. Prior to correlation, all of the luminescence signals were normalized to 10 µM forskolin^3^.

***Apoptosis staining under microscopic conditions.*** Apoptosis was measured using the Annexin-V FITC Apoptosis Detection Kit (Sigma Aldrich), according to the manufacturer’s instructions. Briefly, cells were incubated in cell culture flasks at a density of 1 x 10^5^ cells/mL in RPMI-1640 containing 10% FBS, 5% GlutaMAX, 100 units/mL penicillin/streptomycin with 10 µM, 30 µM, 100 µM, 300 µM, 700 µM or 1 mM odorants for 1 h. Subsequently, the cells were washed in PBS-/- and incubated for 5 minutes with Annexin V and propidium iodide (1:1) in the dark. The cells were gently placed on a 35-mm cover slip and were investigated under a Zeiss Axioskop-2 microscope (Carl Zeiss, Oberkochen, Germany)^4,5^. The Annexin V FITC Apoptosis Detection Kit enabled us to differentiate among early apoptotic cells (green staining), necrotic cells (red staining) and viable cells (no staining).

***Immunocytochemistry.*** For immunocytochemistry experiments, custom-designed antibodies were used against the C-terminus of the OR2AT4, polyclonal, dilution 1:50 (Eurogentec, Seraing, Belgium)^3^; anti-OR51B5, polyclonal, dilution 1:25 (Eurogentec); and anti-caspase-3, polyclonal, dilution 1:300 (Sigma Aldrich). Briefly, 5 mm cover slips were coated for 15 minutes with poly-L-lysine (Sigma Aldrich) and were dried for 30 minutes in the air. We used 60 µl of 1 - 5 x 10^6^/mL K562 cells and incubated them in medium on coated 5 mm cover slips. The medium was removed, and the cells were fixed in 4 % paraformaldehyde for 20 minutes at 4 °C. To wash and permeabilize the membrane, PBS medium including 0.1% Triton X-100 (Sigma Aldrich) (PBST) was used. The K562 cells were incubated with primary antibodies and were diluted in blocking solution, containing PBST, 1% fish gelatin, and 3% horse serum, for 3 h at room temperature. After rewashing three times with PBST, the cells were incubated with a secondary goat anti-rabbit antibody (1:1000) (Alexa Fluor 488, Life Technologies) and 4’6’-diamidino-2-phenylindole (1:300) in blocking solution for 1 h at room temperature. Then, the cells were washed with PBST again and were covered on an object slide with ProLong Gold antifade reagent (Invitrogen). Images were obtained using a Zeiss LSM 510 META confocal microscope (Carl Zeiss, Germany).

***Calcium imaging.*** K562 cells were incubated for 20 minutes in poly-L-lysine-coated (Sigma Aldrich) 35-mm dishes with RPMI-1640 (Life Technologies), 10% FBS, 5% GlutaMAX, and 100 units/mL penicillin/streptomycin, and they were incubated for 20 more minutes in RPMI-1640 containing 3 mM Fura-2-AM (Molecular Probes, Eugene, OR, USA) at 37 °C in 6% CO_2_. White blood cells from AML patients were incubated for 20 minutes in concanavalin A-laminated (Sigma Aldrich) 35-mm dishes. Before measuring the cells, the RPMI-1640 medium was replaced with Ringer’s solution (140 mM NaCl, 5 mM KCL, 2 mM CaCl_2_, 1 mM MgCl_2_, and 10 mM HEPES; pH 7.3). Calcium-imaging experiments were performed as described previously^6,7^. To create Ca^2+^-free conditions, we added 10 mM EGTA to a Ca^2+^-free Ringer’s solution. We used the adenylate cyclase inhibitors MDL-12,330A (10 µM) and SQ-22536 (10 µM), the phospholipase C inhibitor U-73122 (30 µM), and the PKA inhibitor H89 (10 µM) to analyze the OR2AT4-mediated signaling pathway (Sigma Aldrich). Furthermore, we used different calcium channel blockers, such as mibefradil (10 µM), NNC-550396 (30 µM) to inhibit T-type calcium channels and L-cis diltiazem (150 µM) to block CNGA1 and CNGA3 channels as well as L-type calcium channels (Sigma Aldrich). Before and after applying the odorant with the appropriate inhibitor, the cells were pre- and post-incubated with the blocking solution. For the OR2AT4 antagonist experiments, we used Phenirat (300 µM) at the same concentration as Sandalore (300 µM). To investigate cell viability, 100 µM ADP was applied to the cells at the end of the measurements. In previous studies, ADP has been shown to increase the amount of intracellular Ca^2+^ in K562 cells^8,9^.

***Western blotting analysis.*** K562 cells were treated with 100 µM Sandalore in a time-dependent manner (5’, 15’, 30’, and 60’) at 37 °C in 6 % CO_2_. Cells were harvested and homogenized in lysis buffer (RIPA: 50 mM Tris-HCl, 150 mM NaCl, 1 mM EDTA, 1 % Triton X-100, pH 7.4) with protease (cOmplete^TM^, Roche Applied Science, Basel, Switzerland) and phosphatase inhibitors (PhosSTOP, Roche Applied Science). To isolate proteins out of the cell membranes, the samples were ultracentrifuged at the maximum (100,000 g, 1.5 h, 4 °C) after homogenization. All of the Western blot experiments were performed as described elsewhere^3,6^. Detection was performed using the ECL^TM^ Select Western Blotting Detection System (Amersham Biosciences, GE, Healthcare, Solingen, Germany). We used the polyclonal rabbit antibody against p44/42 MAPK (Cat. No. #9102), p38-MAPK (Cat. No. #9212), JNK-SAPK (Cat. No. #9252) and Akt (Cat. No. #4059), as well as the phosphorylated antibody pp44/42-MAPK (Cat. No. #9101), pp38-MAPK (Cat. No. #9211), pJNK-SAPK (Cat. No. #9251), and pAkt (Cat. No. #4060) (Cell Signaling Technology). For the detection of OR2AT4, we used the same α-OR2AT4 antibody as described elsewhere ^3^. For quantification we used the Java-based software ImageJ, version 1.46, to calculate the relative pixel intensity^10^. Inhibitors of the mitogen activated-protein kinase pp44/42-MAPK, U0126 10 µM (Cell Signaling Technology, New England Biolabs), the pp38-MAPK inhibitor SB203580 (10 µM) (Cell Signaling Technology), and the Akt inhibitor LY294002 (10 µM) were used for the blocking experiments (Sigma Aldrich). To investigate the influence of the intracellular Ca^2+^ increase, we inhibited calmodulin kinase II (CaMKII) with KN-62 (10 µM) (Sigma Aldrich). KN-62 binds directly to the CaMKII, leading to consequent inactivation ^11^. Because CaMKII is activated by Ca^2+^, and it is necessary for the activation of calmodulin, one could observe the influence of increased intracellular Ca^2+^. Therefore, we pre-incubated the cells with KN-62 for at least 60 minutes, before starting the Sandalore incubation. The blocking effects on Sandalore-treated cells were observed for a further 60 minutes, before isolating the proteins. To load equal amounts of protein, we calculated the total amount with Bradford (Life Technologies) and optimized it with a Coomassie staining on an SDS-PAGE. For some experiments, we used the antibody against the housekeeping protein vinculin (Cell Signaling Technology) as a control.

***Statistical analysis.*** Statistical analyses were performed with Microsoft Excel software and Sigma Plot software, version 12. All of the results were tested for normality and equal variance. Significance levels were calculated with the two-tailed unpaired t-test, while significant values were categorized as < 0.05 (*), < 0.01 (**), or < 0.0005 (***). Dose-response curves and EC_50_ values were determined with the 4-parameter Hill model. Values are presented as the mean ± SEM (standard error of the mean) of at least three independent experiments.

***Supplementary Figures:***


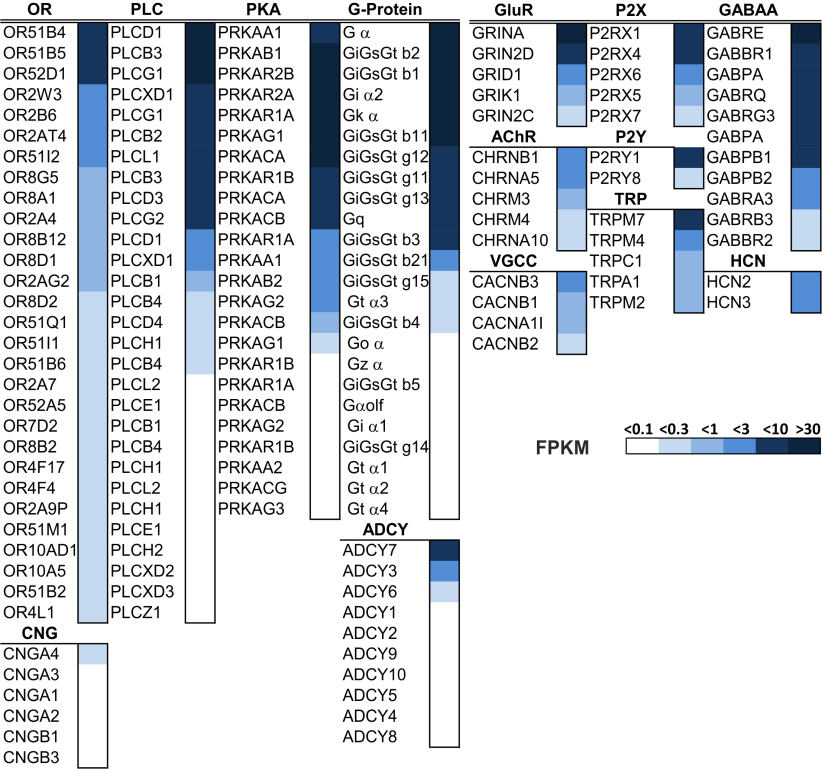


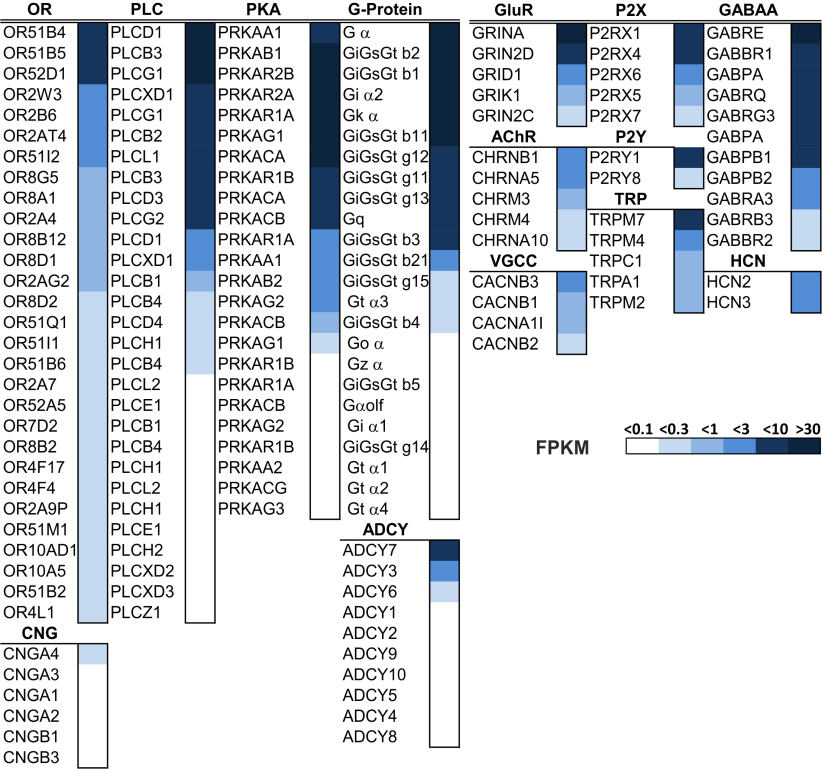


**Supplementary Figure 1.** Expression strength is shown for the detected olfactory receptors and the differently expressed GPCR signaling molecules (phospholipase C: PLC; protein kinase A: PKA; adenylate cyclase: ADCY) in FPKM (fragments per kilobase of exon per million fragments mapped). Transcripts detected at less than 1 FPKM are weak to non-expressed, while transcripts being detected at greater than 1 FPKM are represented as medium to strong.


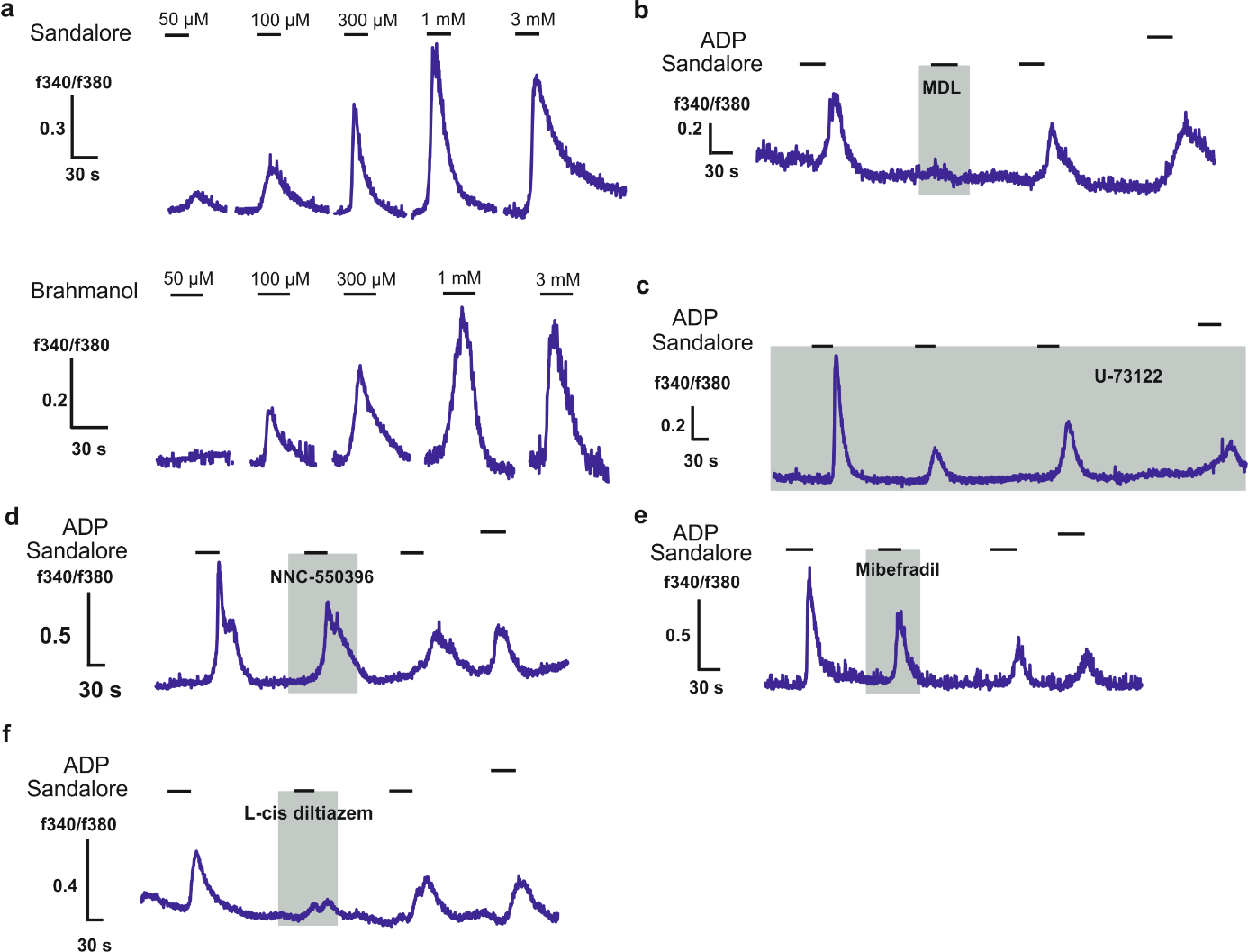


**Supplementary Figure 2. (a)** Sandalore (n=684) and Brahmanol (n=380) increased intracellular Ca^2+^ during application in K562. **(b)** MDL-12,330A (n=258) completely blocked the Sandalore-induced increase in intracellular Ca^2+^. **(c)** U-73122, compared to the control 1^st^ Sandalore-induced (Figure 2d) response could not block (n=177). **(d)** NNC-550396 was not able to decrease the influx of extracellular Ca^2+^ (n=92). **(e)** Mibefradil could not block the influx of extracellular Ca^2+^ (n=67). **(f)** L-cis diltiazem slightly blocked the Ca^2+^ influx (n=182).


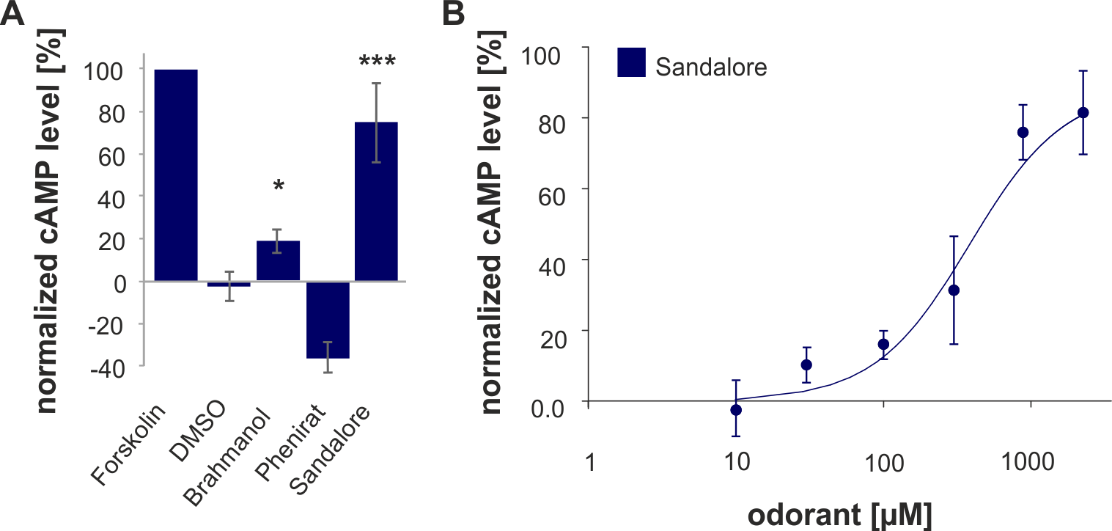


**Supplementary Figure 3.** To investigate the influence on the cAMP-level in K562, we normalized to the positive control forskolin (10 µM). Sandalore and Brahmanol could both significantly increase the cAMP-level in K562 (n=5). Phenirat could not raise the cAMP-level (n=3). Dose response curve for the OR2AT4 ligand Sandalore is shown (EC_50_=310 µM±25).


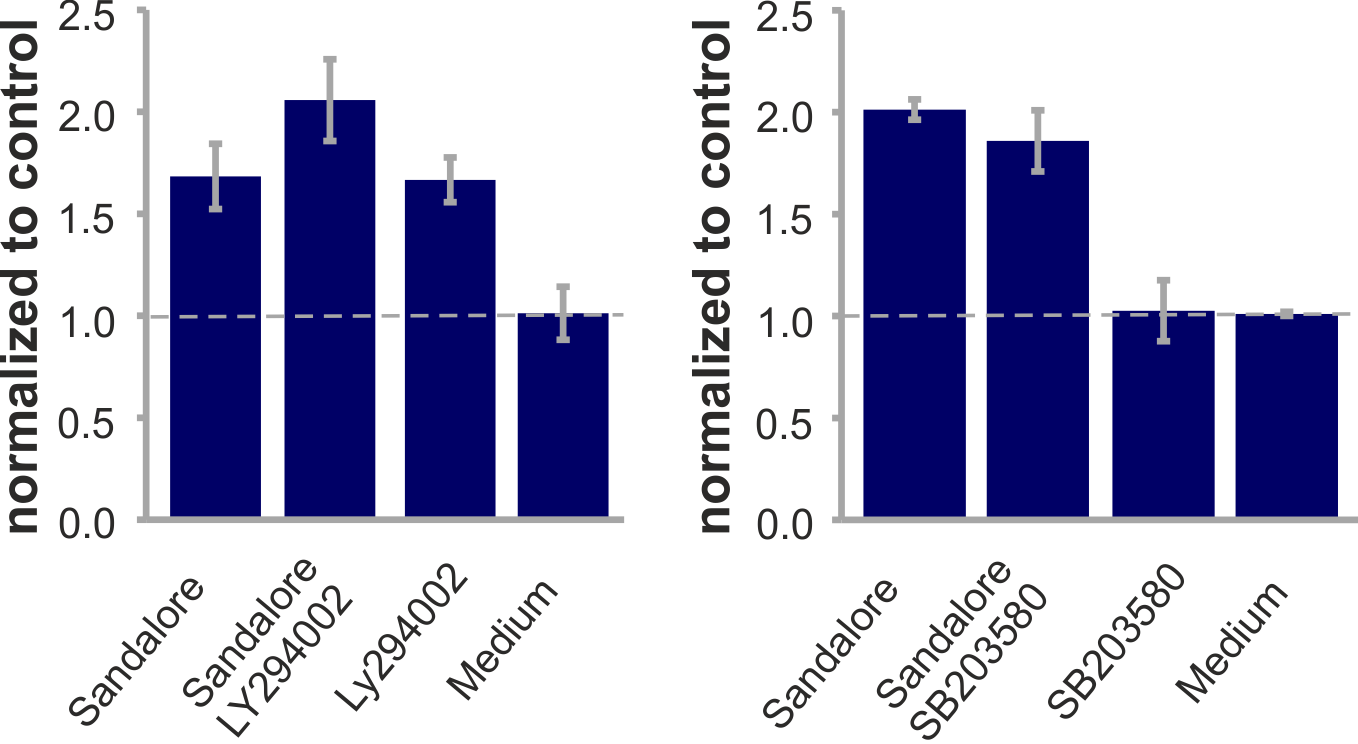


**Supplementary Figure 4.** Akt and p38-MAPK are not necessary for the Sandalore-induced apoptosis. The pp38-MAPK inhibitor SB203580 and Akt inhibitor LY294002 did not affect the Sandalore-induced apoptosis rate (n=8).

**Supplementary Table**

**Supplementary Table 1. Patient characteristics**

|  | Newly diagnosed AML | Relapsed AML |
| --- | --- | --- |
| No. of patients | 10 | 4 |
| Cytogenetic risk group |  |  |
| Favorable | 0 | 1 |
| Intermediate | 7 | 3 |
| Unfavorable | 3 | 0 |
| Age (years) | 66^*^  (29 – 80)^*^ | 49  (37 – 72) |
| White blood cell count (per nL) | 37.7  (9.6 – 184.6) | 21.8  (14.0 – 69.7) |
| Percent blast cells (%) | 72  (53 – 93) | 52  (13 – 74) |
| Blast cell count (per nL) | 28.4  (5.1 – 166.2) | 14.3  (1.8 – 30.7) |

^*^ Median (range)

**References**

1. Trapnell C, Pachter L, Salzberg SL. TopHat: discovering splice junctions with RNA-Seq. *Bioinformatics*. 2009;25(9):1105-1111.

2. Flegel C, Manteniotis S, Osthold S, Hatt H, Gisselmann G. Expression profile of ectopic olfactory receptors determined by deep sequencing. *PLoS ONE*. 2013;8(2):e55368.

3. Busse D, Kudella P, Grüning N, et al. A Synthetic Sandalwood Odorant Induces Wound-Healing Processes in Human Keratinocytes via the Olfactory Receptor OR2AT4. *J. Invest. Dermatol.* 2014.

4. Wang CL, Ng TB, Yuan F, Liu ZK, Liu F. Induction of apoptosis in human leukemia K562 cells by cyclic lipopeptide from Bacillus subtilis natto T-2. *Peptides*. 2007;28(7):1344-1350.

5. Wang CL, Ng TB, Cao XH, Jiang Y, Liu ZK, Wen TY *et al*. CLP induces apoptosis in human leukemia K562 cells through Ca(2+) regulating extracellular-related protein kinase ERK activation. *Cancer Lett.* 2009;276(2):221-227.

6. Neuhaus EM, Zhang W, Gelis L, Deng Y, Noldus J, Hatt H. Activation of an olfactory receptor inhibits proliferation of prostate cancer cells. *J. Biol. Chem.* 2009;284(24):16218-16225.

7. Spehr M, Gisselmann G, Poplawski A, Riffell JA, Wetzel CH, Zimmer RK *et al*. Identification of a testicular odorant receptor mediating human sperm chemotaxis. *Science*. 2003;299(5615):2054-2058.

8. Murgo AJ, Sistare FD. K562 leukemia cells express P2T (adenosine diphosphate) purinergic receptors. *J. Pharmacol. Exp. Ther.* 1992;261(2):580-585.

9. Palme D, Misovic M, Schmid E, Klumpp D, Salih HR, Rudner J *et al*. Kv3.4 potassium channel-mediated electrosignaling controls cell cycle and survival of irradiated leukemia cells. *Pflugers Arch.* 2013;465(8):1209-1221.

10. Schneider CA, Rasband WS, Eliceiri KW. NIH Image to ImageJ: 25 years of image analysis. *Nat. Methods*. 2012;9(7):671-675.

11. Okazaki K, Ishikawa T, Inui M, Tada M, Goshima K, Okamoto T *et al.* KN-62, a specific Ca++/calmodulin-dependent protein kinase inhibitor, reversibly depresses the rate of beating of cultured fetal mouse cardiac myocytes. *J. Pharmacol. Exp. Ther.* 1994;270(3):1319-1324.
